# HNRNPH1 is required for rhabdomyosarcoma cell growth and survival

**DOI:** 10.1038/s41389-017-0024-4

**Published:** 2018-01-24

**Authors:** Yanfeng Li, Jesse Bakke, David Finkelstein, Hu Zeng, Jing Wu, Taosheng Chen

**Affiliations:** 1 Department of Chemical Biology and Therapeutics, St. Jude Children’s Research Hospital, Memphis, TN USA; 2 Department of Computational Biology, St. Jude Children’s Research Hospital, Memphis, TN USA; 3 Department of Immunology, St. Jude Children’s Research Hospital, Memphis, TN USA; 4 Present Address: Division of Rheumatology, Department of Medicine, Mayo Clinic, Rochester, MN USA

## Abstract

Rhabdomyosarcoma (RMS) is an aggressive and difficult to treat cancer characterized by a muscle-like phenotype. Although the average 5-y survival rate is 65% for newly diagnosed RMS, the treatment options for metastatic disease are limited in efficacy, with the 5-y survival rate plummeting to 30%. Heterogenous nuclear ribonucleoprotein H1 (HNRNPH1) is an RNA-binding protein that is highly expressed in many cancers, including RMS. To determine the role HNRNPH1 plays in RMS tumorigenesis, we investigated its expression and effect on growth in three cellular models of RMS: RD, RH30, and RH41 cells. Upon knockdown of *HNRNPH1*, growth of all cell lines was reduced, most likely through a combination of apoptosis and cell cycle arrest. We then recapitulated this finding by performing in vivo xenograft studies, in which knockdown of *HNRNPH1* resulted in a reduction of tumor formation and growth. We used RNA sequencing to identify changes in gene expression after *HNRNPH1* knockdown and found altered splicing of some oncogenes. Our data contribute to understanding the role of HNRNPH1 in RMS development.

## Introduction

Proper regulation of alternative mRNA splicing is paramount for healthy tissue development and maintenance^1^. Alternative splicing allows a relatively small number of genes to produce a very large number of gene products, and it is estimated that 92–94% of genes undergo alternative splicing events^2^. Additionally, alternative splicing events are regulated in a tissue-specific manner that is not entirely understood^3^.

Heterogeneous nuclear ribonucleoprotein H1 (HNRNPH1) is a member of the heterogeneous nuclear ribonucleoprotein family of multifunctional proteins, which are involved in pre-mRNA splicing and mRNA trafficking and stability^4^. HNRNPH1 is primarily localized in the nucleus and has recently been shown to contribute to the development of several types of tumors^5–7^. Of note, HNRNPH1 is required to retain the exon^8^ breakpoint during the processing of *EWS-FLI1* fusion transcripts, which is a driving event in Ewing sarcoma^8^. However, the role of HNRNPH1 in other childhood cancers is not well studied, particularly in the most common childhood soft-tissue sarcoma rhabdomyosarcoma (RMS).

RMS tumors consist of myogenic differentiation features but lack terminally differentiated markers. RMS can be divided into two predominant histopathologic subtypes: embryonal RMS (ERMS) and alveolar RMS (ARMS). Most ARMS tumors express a fusion transcription factor consisting of the DNA binding domain of a PAX family member (i.e., *PAX3* or *PAX7*) and the transactivation domain of *FOXO1* (i.e., *PAX3-FOXO1* or *PAX7-FOXO1*)^9^. RMS purportedly arises from muscle progenitor cells, although mesenchymal progenitor cells and satellite cells may represent other cellular origins. Similar to that of healthy muscle precursor cells, RMS cells express myogenic transcription factors such as *PAX3*, *PAX7*, *MYOD*, *MYF5*, and *MYOG*^10^.

Approximately 350 cases of RMS are diagnosed every year in the United States, and the current therapy usually entails tumor resection and a chemotherapy regimen. Yet, despite advances in treatment, the survival rate of children with RMS is still poor^11^. New therapy for RMS is urgently needed, and identification and validation of novel therapeutic targets represent the critical first steps in the development of new therapies. Here, we investigated the role of HNRNPH1 in regulating RMS growth and survival. Our in vitro and in vivo findings indicate that genetic downregulation of *HNRNPH1* leads to inhibition of RMS cell and tumor growth. Our gene expression and splicing data indicate that HNRNPH1 regulates the expression and splicing of genes, including those involved in cell cycle regulation. Together, our data contribute to understanding the role of HNRNPH1 in RMS development.

## Results

### Highly expressed HNRNPH1 is required for RMS cell growth

**Fig. 1:**
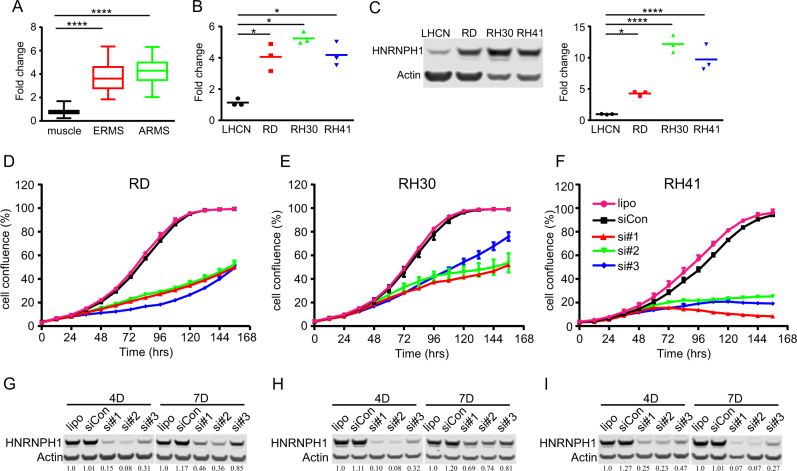
*HNRNPH1* is highly expressed and required for RMS cell growth. **a** Microarray data generated from a survey of RMS primary tumors by Williamson et al. ^14^, in addition to healthy human quadriceps (muscle) data, were used to determine the fold change in *HNRNPH1* expression (muscle, *n* = 40; ERMS, *n* = 32; ARMS, *n* = 57). **b** Quantitative RT-PCR of *HNRNPH1* expression in LHCN-M2 (LHCN) and RMS cells (RD, RH30, and RH41). Data are expressed relative to LHCN cells as the mean ± SD (*n* = 3). **c** Immunoblot analysis and quantification of whole-cell lysates prepared from LHCN and RMS cells detected with antibodies against HNRNPH1 and β-actin (Actin, loading control). Data were quantified with ImageJ software (mean ± SD, *n* = 3). **d–f** Cell growth curves of lipofectamine alone (lipo), siControl (siCon), and si#1–3 (3 individual *HNRNPH1* siRNAs) were obtained by using the Incucyte Zoom live-cell imaging system and data are expressed as cell confluence (%; mean ± SD, *n* = 2). **g–i** Immunoblot analysis of whole-cell lysates prepared from *HNRNPH1* siRNA–transfected **g** RD, **h** RH30, and **i** RH41 cells with antibodies against HNRNPH1 and β-actin. HRNRPH1 quantification is shown below gels and normalized to actin; relative intensity for the sample treated with lipo alone was set as 1.0. *P < 0.05; **P < 0.01; ***P < 0.001; ****P < 0.0001, all data were compared to the control group using one-way ANOVA

*HNRNPH1* is overexpressed in various human cancers, including hepatocellular, pancreatic, and laryngeal carcinomas^5–7,12,13^. However, the role HNRNPH1 plays in RMS has not been studied. Analysis of data produced from a survey of RMS primary tumors conducted by Williamson et al.^14^ revealed that *HNRNPH1* is overexpressed in ARMS and ERMS tumors, when compared with that in healthy human quadriceps muscle (Fig. 1a). Furthermore, *HNRNPH1* mRNA and its protein were highly expressed in the RD, RH30, and RH41 RMS cell lines (Fig. 1b and c). The elevated expression of *HNRNPH1* was also observed in a panel of RMS patient derived xenografts (PDX) (Supplementary Fig. S1)^15–17^. To study the function of HNRNPH1 in RMS cells, we used RNAi to knockdown (KD) endogenous *HNRNPH1*. By using three individual siRNAs (si#1–3) targeting *HNRNPH1*, we observed that KD of *HNRNPH1* in RD, RH30, and RH41 cells decreased growth when compared with that of the transfection reagent alone or nontargeting siRNA (siControl) controls (Fig. 1d–f). We confirmed the KD efficiency by immunoblot analysis (Fig. 1g–i), noting that si#1 and si#2 had similarly higher KD efficiency. Because of this, we used si#1 and si#2 in subsequent studies.

### Knockdown of HNRNPH1 leads to cell cycle arrest and cell death

**Fig. 2:**
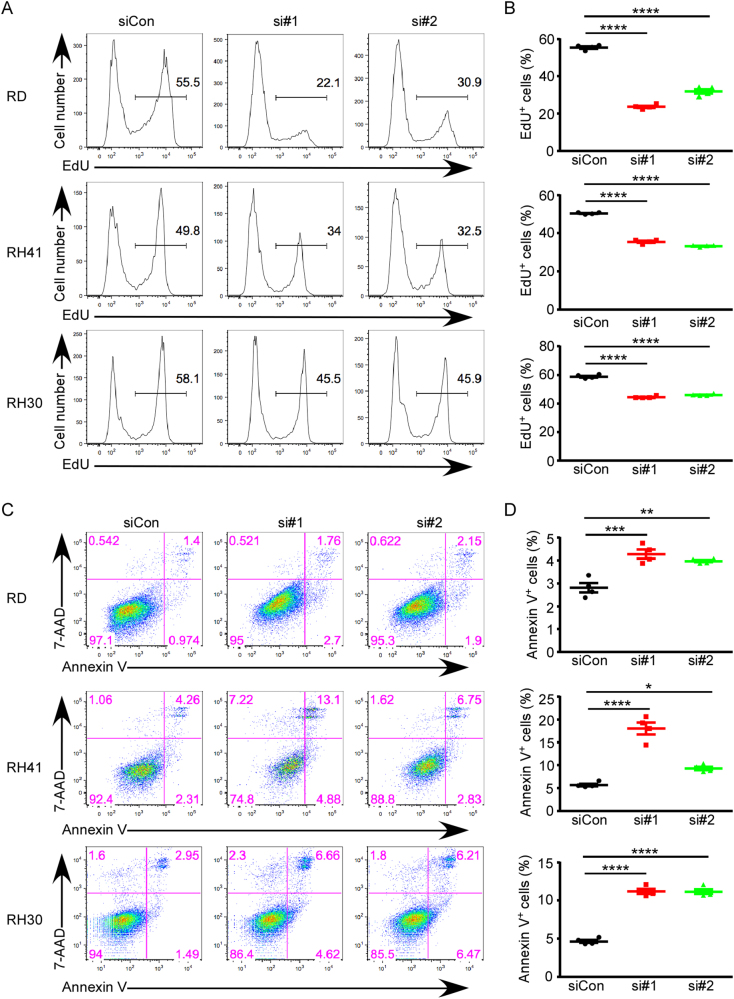
*HNRNPH1* knockdown leads to cell cycle arrest and apoptosis. **a** Flow cytometric analysis of EdU incorporation 48 h after *HNRNPH1* siRNA transfection in RMS cells. RD cells were labeled with EdU for 3 h. RH30 and RH41 cells were labeled with EdU for 2 h. **b** The percentage of EdU^+^ cells were quantified (mean ± SD, *n* = 4). **c** Flow cytometric analysis of Annexin V^+^ cells 96 h after *HNRNPH1* siRNA transfection in RMS cells. **d** The percentage of Annexin V^+^ cells were quantified (mean ± SD, *n* = 4). *P < 0.05; **P < 0.01; ***P < 0.001; ****P < 0.0001

We hypothesized that *HNRNPH1* KD attenuated cell growth by causing cell cycle arrest and apoptosis, as we did not observe an obvious reduction in cell size (Supplementary Fig. S2A). Interestingly, gene ontology (GO) analysis revealed that RH30 and RH41 cells appear to undergo muscle differentiation to a certain degree (Supplementary Fig. S2B). To test whether the cells undergo cell cycle arrest and apoptosis, we analyzed EdU incorporation and Annexin V staining. EdU is a nucleoside analog of thymidine and is incorporated into DNA during active DNA synthesis. EdU incorporation, therefore, directly measures active DNA synthesis during the S-phase of the cell cycle. As shown in Fig. 2a, KD of *HNRNPH1* in RMS cells decreased the percentage of EdU^+^ cells, which confirmed that *HNRNPH1* KD decreased proliferation of RMS cells (Fig. 2b). To determine whether *HNRNPH1* KD also induces cell death, we stained the cells with fluorescently labeled Annexin V and performed flow cytometric analysis (Fig. 2c). Indeed, *HNRNPH1* KD increased the percentage of Annexin V^+^ apoptotic cells (Fig. 2d). Interestingly, apoptosis induction was mild in RH30 and RH41 cells, and milder in RD cells, yet the inhibition of proliferation by *HNRNPH1* KD was the strongest in these cells, suggesting that HNRNPH1 most likely functions through different pathways in different RMS subtypes or cells.

### Knockdown of HNRNPH1 inhibits cell proliferation and activates apoptosis through CDK2/4/6 expression and cleavage of PARP and caspase-3

**Fig. 3:**
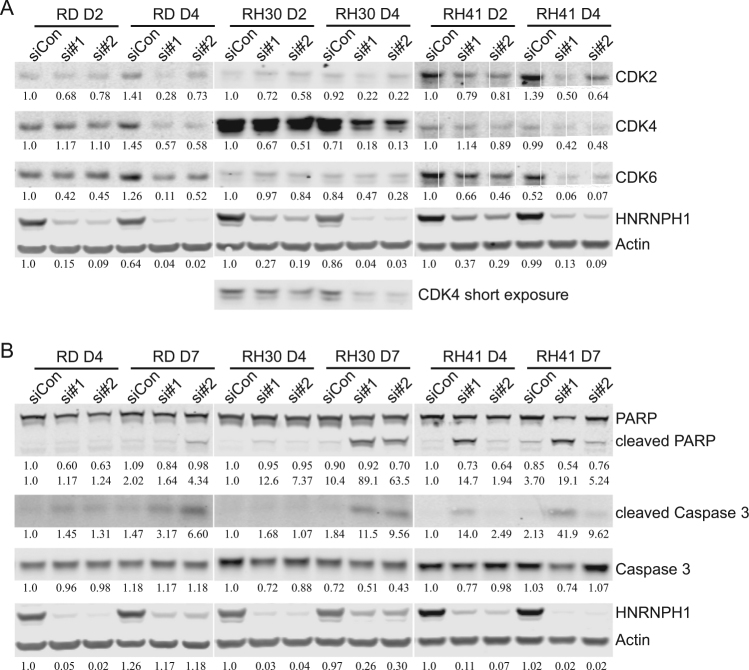
Knockdown of *HNRNPH1* leads to decreased CDK2/4/6 protein levels and elevated apoptosis markers. **a** Immunoblot analysis of whole-cell lysates prepared from RMS cells (RD, RH30, and RH41) 2 and 4 days post-*HNRNPH1* siRNA transfection with antibodies against CDK2/4/6, HNRNPH1, and β-actin. **b** Immunoblot analysis of whole-cell lysates prepared from RMS cells (RD, RH30, and RH41) 4 and 7 days post-*HNRNPH1* siRNA transfection with antibodies against PARP, cleaved PARP, caspase-3, cleaved caspase-3, HNRNPH1, and β-actin. All quantification is shown below the gels and normalized to actin. CDK4 was quantified using the short exposure for RH30 cells

Downregulation of *HNRNPH1* caused cell cycle arrest (Fig. 2). We also noted that CDK2/4/6 transcript levels are decreased in RNA-seq analysis (Supplementary Dataset 1), and that *NUCKS1*, a known CDK target, was among the genes most downregulated by HNRNPH1siRNA in all three cell lines (Supplementary Fig. S2C-D). These data prompted us to further examine the regulation of known cell cycle genes. Quantitative RT-PCR (Supplementary Fig. S3A-C) and immunoblotting (Fig. 3a, b) confirmed the downregulation of *CDK2*, *CDK4*, and *CDK6* mRNA and their proteins by *HNRNPH1* siRNAs. In RD cells, the protein levels of CDK2/4/6 were all decreased by 4 days post *HNRNPH1* siRNA transfection (Fig. 3a). In RH30 cells, CDK4 levels were markedly high and obviously decreased by *HNRNPH1* siRNAs. In contrast with RH30 cells, RH41 cells had lower levels of CDK4 but higher levels of CDK2 and CDK6, and CDK2 and CDK6 were clearly decreased by *HNRNPH1* siRNAs. These results are consistent with the observation that *HNRNPH1* KD inhibits cell cycle progression.

To further investigate the mechanism in which *HNRNPH1* KD promotes apoptosis (Fig. 2c and d), we examined the levels of cleaved PARP and caspase-3, which are apoptosis makers. As shown in Fig. 3b, the levels of cleaved PARP and caspase-3 mildly increased upon *HNRNPH1* KD. Together, these data indicate that downregulation of *HNRNPH1* inhibits cell proliferation and mildly induces apoptosis.

### Knockdown of HNRNPH1 inhibits RMS xenograft growth in vivo

**Fig. 4:**
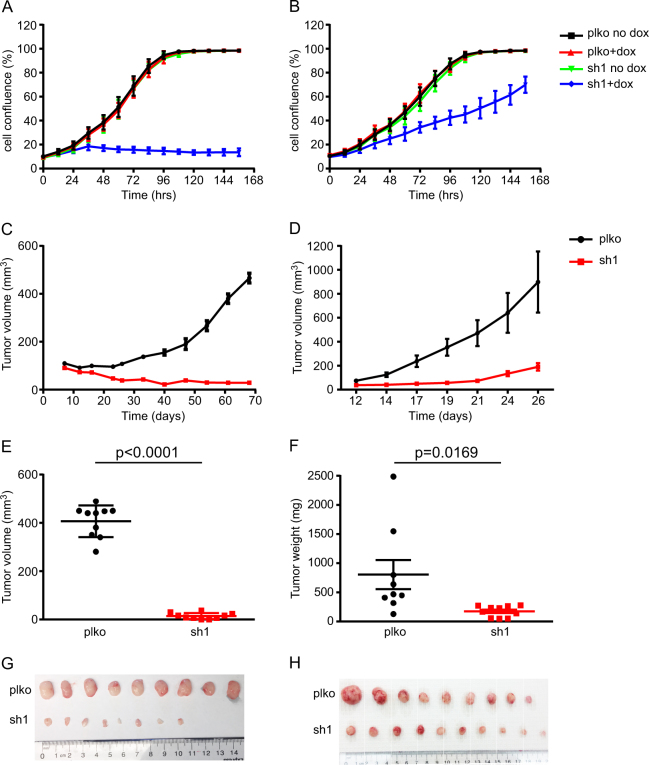
Loss of *HNRNPH1* inhibits tumor growth. **a** RD and **b** RH30 Tet-On *HNRNPH1* shRNA stable cell lines were induced with 200 ng/mL doxycycline (dox). Incucyte Zoom was used to quantify cell growth, and data are expressed as percentage of cell confluence (%; mean ± SD, *n* = 2). **c**, **d** Mice were treated with 0.4 mg/mL dox in their drinking water immediately after cell transplantation. **c** RD and **d** RH30 xenograft volumes were measured at the indicated days after cell transplantation. **e**, **g** RD and **f**, **h** RH30 xenografts were collected and weighed at the end of the experiments. plko, Tet-pLKO-puro vector control; sh1, *HNRNPH1* shRNA#1 in the pLKO-Tet-On vector

To determine whether *HNRNPH1* KD also inhibits RMS tumor growth in vivo, we generated doxycycline-inducible *HNRNPH1* shRNA stable RD and RH30 cells. Inducible KD of *HNRNPH1* inhibited cell growth in vitro (Fig. 4a, b) to levels nearly identical as those elicited by the siRNAs (Fig. 1). Of note, control cells (sh#1 without doxycycline) exhibited identical growth patterns as cells transduced with empty vector (plko). We confirmed the inducible KD efficiency by immunoblot analysis (Supplementary Fig. S4A and S4B). To study the tumor growth in vivo, we subcutaneously injected both doxycycline-inducible control (pLKO empty vector) and sh#1 (pLKO-Tet-On-*shHNRNPH1*) cells into the opposing flanks of *NU/NU* mice. The mice were randomly assigned into two groups: (1) doxycycline treatment immediately after cell transplantation and (2) doxycycline treatment after the tumors became palpable. In Group 1, inducing *HNRNPH1* KD immediately after cell transplantation dramatically inhibited tumor growth of both RD (Fig. 4c) and RH30 cells (Fig. 4d), consistent with the growth inhibition observed in vitro. Note that the pattern of growth inhibition in vitro is not identical to that in vivo, likely because of the different experimental setting including time course. When we excised the tumors and assessed their overall volumes, we found that the tumors with *HNRNPH1* KD were much smaller than the controls (Fig. 4e–h).

To determine whether *HNRNPH1* KD reduces the tumor burden of mice with existing tumors, we treated a cohort of mice with doxycycline after their tumors were palpable (Group 2). As shown in Supplementary Figs. S4C (RD) and S4D (RH30), tumor growth was indeed inhibited after doxycycline treatment. However, we observed that the tumors regained their grow kinetics after a prolonged time of doxycycline treatment. This is likely due to various possible mechanisms which may include loss of *shHNRNPH1*-expressing cells. Indeed, we found that *HNRNPH1* levels were not downregulated in terminal tumor samples after a prolonged time of doxycycline treatment (Supplementary Fig. S4G and S4H). To confirm that *HNRNPH1* KD reduces the tumor burden we treated a second cohort of mice with doxycycline after their tumors were palpable and examined the relationship between tumor volumes and protein levels of HNRNPH1 at earlier time points after doxycycline treatment. We showed that at an early time-point (day 17, which is 5 days after doxycycline treatment), reduced tumor growth (Supplementary Fig. S4I) corresponds to knockdown of *HNRNPH1* (Supplementary Fig. S4J). This is highly suggestive that *HNRNPH1* KD results in cellular death, but cells with low sh*HNRNPH1* expression (and thus higher *HNRNPH1* expression) are able to repopulate the tumor after a prolonged period of time. Together, these data reveal an association between the levels of *HNRNPH1* expression and tumor growth in vitro and in vivo, suggesting that downregulating *HNRNPH1* may inhibit RMS growth.

### Knockdown of HNRNPH1 changes global gene expression related to cell growth

**Fig. 5:**
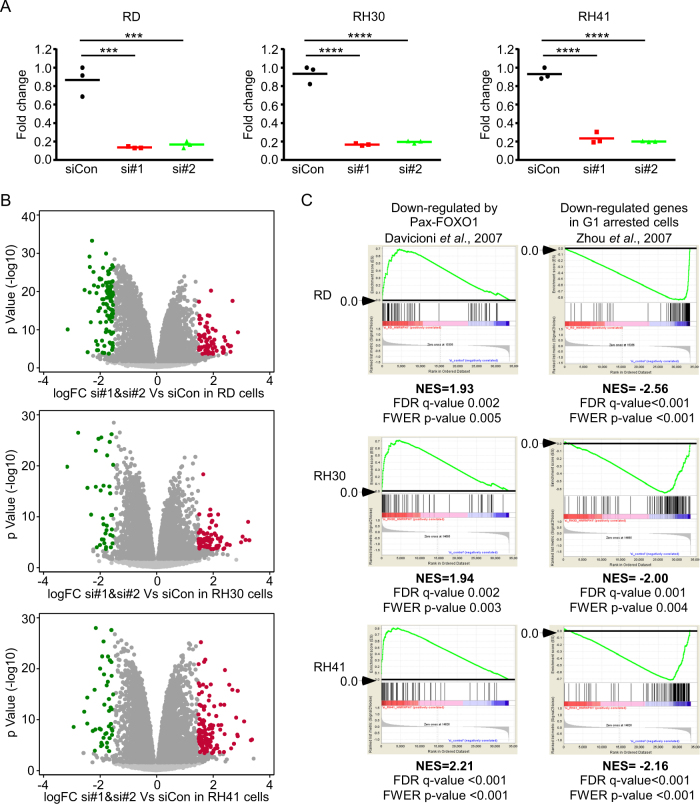
*HNRNPH1* knockdown leads to a global transcriptome change in RMS cells. **a** Quantitative RT-PCR of *HNRNPH1* expression in *HNRNPH1* siRNA-transfected RMS cells (48 h). **b** Volcano plots of two individual *HNRNPH1* siRNAs vs. siCon in RD, RH30, and RH41 cells. Genes with *P* > 3 (–log10) and absolute logFC < 1.5 are highlighted in green (downregulated) or red (upregulated). Dark grey dots indicate genes with a false discovery rate <5%. **c** Gene set enrichment analysis of the highlighted genes in **b**. Genes that were upregulated by *HNRNPH1* siRNAs were compared with those downregulated by PAX-FOXO1, as described in Davicioni et al. 2007 [18]. Genes downregulated by *HNRNPH1* siRNAs were compared with those downregulated in G1-arrested cells, as described in Zhou et al. 2007 [19]

To further understand the roles that HNRNPH1 plays in regulating RMS cell growth and survival, we used RNA-seq to examine the effect of *HNRNPH1* KD in RD, RH30, and RH41 cells on global gene expression. The experiments were performed in triplicate with the si#1 and si#2 *HNRNPH1* siRNAs. We confirmed the KD efficiency of the siRNAs by quantitative RT-PCR (Fig. 5a). RNA-seq data were analyzed to identify the genes with altered expression in response to *HNRNPH1* KD (Supplementary Dataset 1). We observed several genes with significantly increased or decreased fold change (FC) expression levels (absolute logFC > 1.5, *P* > 3.0 [–log10]) (Fig. 5b; downregulated genes denoted in green and upregulated genes in red). We next performed a gene set enrichment analysis of the statistically upregulated and downregulated genes (Fig. 5c and Supplementary Fig. S5), which showed that *HNRNPH1* KD—upregulated genes were highly similar to the genes downregulated by PAX-FOXO1 fusion proteins^18^. The PAX-FOXO1 fusion is oncogenic and promotes cell growth through dysregulation of its downstream transcriptional targets. Therefore, the observation that the genes downregulated by PAX-FOXO1 were upregulated by *HNRNPH1* KD suggests that these genes play a role in promoting cell cycle arrest or cell death. In contrast, the genes downregulated by *HNRNPH1* siRNAs shared a striking resemblance to the genes downregulated in G1-arrested cells^19^, which further confirmed the phenotypic effect we observed in RMS cell cultures (Fig. 2).

### HNRNPH1 regulates gene splicing in RMS cells

**Fig. 6:**
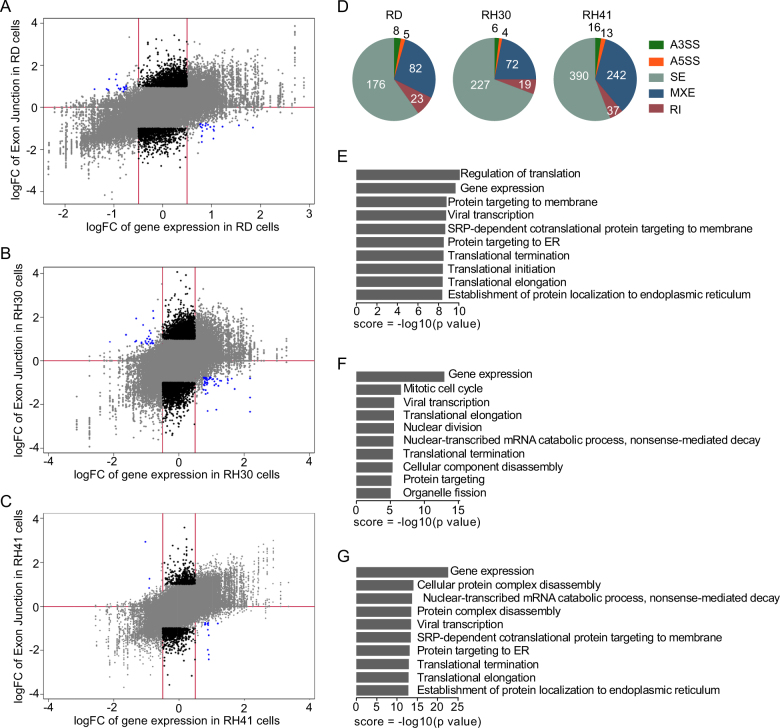
HNRNPH1 regulates gene splicing in RMS. **a**–**c** Two individual siRNAs targeting *HNRNPH1* were compared against control siRNA, with gene expression (logFC) plotted on the X-axis and exon junctions (logFC) on the Y-axis. Significant changes in exon junction expression (|logFC exon junction| >1 and |logFC gene| <0.05) were classified as candidate alternative splicing events (black). **d** Summary of the number and type of statistically significant alternative splicing events, as determined by multivariate analysis of transcript splicing (MATS) (false discovery rate <0.05). A3SS, alternative 3´ ends; A5SS, altered 5´ ends; SE, cassette exons; MXE, mutually exclusive exons; and RI, retained introns. **e**–**g** Bar charts show gene ontology categories significantly enriched (false discovery rate <5%) in the alternatively spliced genes induced by *HNRNPH1* siRNA, as determined by MATS

HNRNPH1 is largely involved in RNA alternative splicing. In an effort to fully understand the role HNRNPH1 plays in RMS tumorigenesis, we investigated the changes in alternative splicing induced by *HNRNPH1* KD. By comparing gene expression data with exon junction data, we determined the alternative splicing changes in RD, RH30, and RH41 cells (Fig. 6a–c and Supplementary Dataset 2). We classified genes with an absolute logFC less than 0.5 as having no overall gene expression change and those with a logFC greater than 0.5 as a gene expression change. We also classified genes with an absolute logFC of exon junctions greater than 1.0 as those with altered splicing upon *HNRNPH1* KD. We specifically analyzed the genes that did not have an overall gene expression change (absolute logFC < 0.5) but did have altered splicing (absolute logFC of exon junctions >1.0) (Fig. 6a–c, black). For a small subset of genes, the overall expression level and exon junction level changed in opposing directions in response to *HNRNPH1* KD (Fig. 6a–c, blue).

To quantify the alternative splicing events in response to *HNRNPH1* KD, we performed MATS on the candidate genes and found that the overall pattern was similar among the three RMS cell lines. The predominant splicing events were cassette exons and mutually exclusive exons. Additionally, we performed GO analysis of the candidate alternatively spliced genes and found that these genes consisted largely of transcriptional targets that affect gene expression (Fig. 6d–g). To confirm the alternative splicing insights derived from the loss of *HNRNPH1*, we specifically examined two highly altered candidate genes: *CTNNB1* and *MDM4*. Indeed, the splicing form of *CTNNB1* switched in response to *HNRNPH1* KD in RMS cells (Supplementary Fig. S6A–E). *MDM4* splicing was also affected by *HNRNPH1* KD, resulting in inclusion of intron 1 (Supplementary Fig. S6F, G). Of note, although *CTNNB1* and *MDM4* both underwent a splicing junction change, the resulting protein sequence was conserved, suggesting that the protein function of these genes is unaffected^20, 21^.

## Discussion

HNRNPH1 is primarily known for its role in regulating alternative splicing events. We confirmed the role of HNRNPH1 in regulating the alternative splicing of such genes as *CTNNB1* and *MDM4*. It is interesting to note that although the alternative splicing of *CTNNB1* and *MDM4* was affected by *HNRNPH1* knockdown, the resultant protein sequence is the same. One explanation would be that only one of the variants of *CTNNB1* or *MDM4* was impacted. Indeed, CTNNB1 has been shown to have altered splice variants expressed in different stages of cancer tissue, although the significance is still under investigation^20,22^. Whether this alternative splicing contributes to the phenotype of HNRNPH1 inhibition in RMS cell proliferation and survival warrants further investigation. Any insight would be important for any cancer type that CTNNB1 or MDM4 plays a physiological role.

In addition, HNRNPH1 has also been shown to have tissue-specific functions^23^ and to positively regulate tumor survival^5,24^. Our studies revealed that HNRNPH1 plays a vital role in regulating RMS cell proliferation and survival. *HNRNPH1* KD in both ERMS (RD) and ARMS (RH30 and RH41) cells decreased cell proliferation and the percentage of the cell population in S phase and mildly increased apoptosis. This observation was recapitulated in vivo by xenograft studies, in which *HNRNPH1* KD resulted in a marked growth reduction in both RD and RH30 cells. These findings suggest that inhibition of HNRNPH1 decreases tumor growth.

Our findings confirm that *HNRNPH1* KD mildly induced apoptosis, in part, through cleaved caspase-3 and PARP activation, which is consistent with previous observations^25^. This also supports the hypothesis that *HNRNPH1* depletion diminishes tumor growth by activating apoptosis pathways. Indeed, HNRNPH1 regulates the splicing of the proapoptotic effector BCL-X^26^ and maintains p53 pre-mRNA 3´-end processing^27^. Splicing of *EWS-FLI1* in Ewing sarcoma has been shown to be regulated by HNRNPH1^8^. ARMS, like some Ewing sarcomas, is driven by a fusion PAX-FOXO1 protein^9^. Although we did not observe any alterations in *PAX-FOXO1* expression or splicing in response to *HNRNPH1* KD, we did note that HNRNPH1 and PAX-FOXO1 regulate the expression of many shared genes^18^. This may be due to inhibition of yet unidentified PAX-FOXO1 regulatory proteins or a global gene profile switch that favors increased cell death in response to *HNRNPH1* KD. This is supported by the fact that *HNRNPH1* KD reduced the expression of genes that are known to be downregulated upon G1 cell cycle arrest^19^. The result of the global gene expression and splicing alterations upon *HNRNPH1* KD is striking.

In our xenograft studies, we found that *HNRNPH1* KD caused a profound defect in tumor formation and growth. These data are consistent with our findings that loss of HNRNPH1 results in decreased cell viability through inhibition of cell cycle and mild activation of apoptosis. Additionally, in established and actively growing tumors, *HNRNPH1* KD initially halted tumor growth; however, the tumors eventually regained their growth kinetics. The tumors likely regained their growth kinetics due to a small subset of cells that escape doxycycline induced inhibition of HNRNPH1. Nevertheless, *HNRNPH1* KD either immediately at tumor implantation or after tumor development significantly reduced tumor growth, suggesting that HNRNPH1 is a valid antitumor therapeutic target for the treatment of RMS.

In summary, we show that HNRNPH1 is vital for tumor survival, most likely through regulation of cell proliferation and cell death. We have shown that both siRNA-mediated and shRNA-mediated downregulation of *HNRNPH1* resulted in profound growth defects in vivo. Lastly, we have shown that *HNRNPH1* KD results in a shift in global gene expression with a high similarity to that observed in G1 arrested cells. With these findings, we add to the growing knowledge that HNRNPH1 plays a prominent role in tumor survival, including that of RMS.

## Materials and methods

### Chemicals and reagents

Fetal bovine serum (FBS) was purchased from Thermo Fisher Scientific (Waltham, MA). Tet System Approved FBS (631106) was purchased from Clontech Laboratories (Mountain View, CA). DMEM (10564), RPMI 1640 (11835), Lipofectamine 3000 and Lipofectamine RNAiMax were purchased from Invitrogen (Carlsbad, CA). Zinc sulfate, vitamin B12, apotransferrin, dexamethasone, and insulin were purchased from Sigma-Aldrich (St. Louis, MO). Hepatocyte growth factor (GF116) was purchased from EMD Millipore (Billerica, MA). Bovine fibroblast growth factor was purchased from BioPioneer Inc. (HRP-0011) (San Diego, CA). Puromycin dihydrochloride (P9620) was purchased from Sigma-Aldrich. Doxycycline hydrochloride (D43020) was purchased from Research Products International (Mount Prospect, IL). *HNRNPH1* siRNAs (#1 s6729; #2 s6730) were purchased from Thermo Fisher Scientific. The #3 *HNRNPH1* siRNA (S104258800) and nontargeting siRNA (1027281) were purchased from Qiagen (Valencia, CA). HNRNPH1 (Hs01033855), CDK2 (Hs01548894), CDK4 (Hs00364847), CDK6 (Hs01026371), and 18S RNA TaqMan probes were purchased from Thermo Fisher Scientific. PowerUp SYBR Green Master Mix (A25777) and TaqMan Fast Advanced Master Mix (Cat. 4444557) were purchased from Thermo Fisher Scientific. HNRNPH1 antibody (NB100-385) was purchased from Novus Biologicals (Littleton, CO). Cleaved PARP (9546), cleaved caspase-3 (9661), caspase-3 (9662), CDK1 (28439), CDK2 (2546), CDK4 (12790), and CDK6 (3136) antibodies were purchased from Cell Signaling Technology (Danvers, MA). The β-Actin antibody (A5441) was purchased from Sigma-Aldrich. The following secondary antibodies were purchased from LI-COR (Lincoln, NE): 800CW goat anti-mouse IgG (925–32210), 800CW goat anti-rabbit IgG (925–322110), 680LT goat anti-rabbit IgG (925–68021), and 680LT goat anti-mouse IgG.

### Cell culture

All cell lines were maintained in a humidified incubator at 37 °C with 5% CO_2_. HSMM and SKMC cells were purchased from Lonza (Allendale, NJ) and cultivated according to manufacturer protocol. LHCN-M2 cells were grown as previously described^28,29^. The RD, RH30, and 293T cell lines were obtained from ATCC (Manassas, VA). RH41 was described previously^30,31^. The cell lines were authenticated by short tandem repeat DNA profiling and routinely tested negative for mycoplasma contamination. The RD, RH30, and RH41 cell lines were maintained in RPMI 1640 containing 100 U/mL of penicillin, 100 mg/mL of streptomycin, and 10% FBS. The 293T cells were maintained in DMEM supplemented with 100 U/mL of penicillin, 100 mg/mL of streptomycin, and 10% FBS. To induce shRNA expression in inducible stable cells, 200 ng/mL of doxycycline was added to the culture medium.

### Studies of cell growth in response to siRNA knockdown of HNRNPH1

Based on sequence, the *HNRNPH1* siRNA is specific for HNRNPH1 and not predicted to target other family members such as HNRNPH2, and as confirmation, the levels of HNRNPH2 was not significantly changed by *HNRNPH1* siRNA as revealed in the RNA-seq data (Supplementary Dataset 1). All siRNAs were dissolved in ultrapure distilled water (Invitrogen) to generate 20-µM stocks. Lipofectamine RNAiMAX was used to transfect siRNA (siRNA stock to Lipofectamine RNAiMAX ratio = 1:1 v/v). After 6 h, the transfection medium was replaced with fresh medium. For cells harvested 2 and 4 days post transfection, 2 × 10^5^ (RD and RH30) or 2.4 × 10^5^ (RH41) cells were seeded per well of 6-well plate, and 2 µL of RNAiMAX and 2 µL of 20 µM siRNA were used for transfection in 2 mL of culture medium. To study cell growth using the Incucyte ZOOM live-cell imaging system (Essen Bioscience, Ann Arbor, MI), 5-ethynyl-2´-deoxyuridine (EdU) incorporation or annexin V staining assays, 1 × 10^5^ (RD and RH30) or 1.2 × 10^5^ (RH41) cells were seeded per well of 6-well plate. After 12 h, 1 µL of Lipofectamine RNAiMAX mixed with 1 µL of 20 µM siRNA was used for transfection in 2 mL of culture medium. For the cell growth studies using the IncuCyte ZOOM system, immediately after replacing the transfection medium with fresh medium,16 photos per well were taken every 12 h for 7 days. The data were analyzed with the IncuCyte ZOOM software.

### Plasmid construction, lentivirus generation, and viral transduction

The doxycycline-inducible *HNRNPH1* shRNA plasmids (containing the same nucleotide sequence as siRNA#1) were constructed as previously described^32,33^. The empty pLKO-Tet-On plasmid (Tet-pLKO-puro, gift from Dmitri Wiederschain, Addgene plasmid #21915) was used as a nontargeting control. Lentiviruses were generated in 293T cells in 6-well plates. We combined 1 µg of human pLKO vector, 0.75 µg of psPAX2 (gift from Didier Trono, Addgene plasmid #12260), and 0.25 µg of pMD2.G (gift from Dider Trono, Addgene plasmid #12259) with 5 µL Lipofectamine 3000 in Opti-MEM for transfection. The transfection medium was replaced with fresh medium after 6 h, and viruses were collected after 48 h. To remove cells and debris, the medium was filtered with a 0.45 µm PES filter and frozen at −80 °C. Viral transduction was accomplished with 1 mL of virus-containing medium mixed with 3 mL of fresh medium added to a 6 cm-dish of RMS cells at 40% cellular confluence with 8 µg/mL Polybrene (Sigma-Aldrich) for 16 h. The viruses were transduced for 1 day, and the transduction medium was replaced with fresh medium containing 0.4 µg/mL puromycin. The cells were grown in culture for 4 days to establish pooled puromycin-resistant stable cells. To maintain the pooled stable cells for future experiments, 0.2 µg/mL of puromycin was used. For induction, the cells were grown in medium with tetracycline-free FBS before adding doxycycline.

### Flow cytometry

To study the effect of *HNRNPH1* siRNA on cell proliferation, we performed EdU incorporation assays with the Click-iT Plus EdU Pacific Blue Flow Cytometry Assay Kit (C10636, Thermo Fisher Scientific). After 48 h siRNA transfection, 20 µM of EdU was added to the culture medium to label the cells (RD for 3 h; RH30 and RH41 for 2 h). Staining was performed as described by the manufacturer. To study the effect of *HNRNPH1* siRNA on apoptosis, we performed annexin V staining assays with the Annexin V-FITC Apoptosis Detection Kit (BMS500FI-300, Thermo Fisher Scientific). After 96 h siRNA transfection, all cells (including cells in the medium) were collected for annexin V staining. The experiment was performed according to manufacturer instructions. The cells were analyzed with a custom-configured BD Fortessa flow cytometer and FACSDiva software (BD Biosciences, San Jose, CA). Data were analyzed with FlowJo software (FlowJO, LLC, Ashland, OR). All experiments were performed with four biologic replicates.

### RNA extraction and quantitative RT-PCR

RNA was extracted with a Maxwell SimplyRNA Tissue Kit and Maxwell instrument (Promega, Madison, WI). RNA concentrations were measured with a NanoDrop 8000 UV–Vis spectrophotometer (Thermo Fisher Scientific). The SuperScript VILO cDNA Synthesis Kit (Life Technologies, Carlsbad, CA) was used to synthesize cDNA. We used 1 µg of RNA for 20 µL of reverse transcription reaction. We then diluted the cDNA to one-fifth of its original concentration and used 1 µL for a 10 µL quantitative RT-PCR reaction. TaqMan probes were used to determine *HNRNPH1*, *CDK2*, *CDK4*, and *CDK6* expression, whereas SYBR Green (Thermo Fisher Scientific) was used to determine *CTNNB1* isoform expression. An ABI 7900HT Fast Real-Time PCR system (Life Technologies) was used in accordance with the TaqMan Fast or SYBR Green standard protocol. The expression of *HNRNPH1*, *CDK2*, *CDK4*, and *CDK6* was normalized to that of the 18S rRNA housekeeping gene. Expression of *CTNNB1* isoforms was normalized to that of *GAPDH*. Each experiment was performed with three biologic replicates. Primers sequences are listed in Supplementary Table S1.

### Immunoblot analysis

The cells in each well of a 6-well plate were lysed with 100 µL of RIPA lysis buffer (Thermo Fisher Scientific). The protein concentration was determined with the Pierce BCA Protein Assay Kit (23225, Thermo Fisher Scientific) and read at 562 nm. Protein samples (15–20 µg/well) were loaded into NuPAGE 4–12% Bis-Tris Midi protein gels (WG1403BOX, Thermo Fisher Scientific). Proteins were transferred to nitrocellulose with an iBlot Gel Transfer system (Thermo Fisher Scientific). Membranes were blocked with Odyssey blocking buffer (LI-COR) for at least 30 min at room temperature. The primary antibodies were diluted in Odyssey blocking buffer to 1:1000 and incubated at 4 °C overnight. Secondary antibodies were diluted in Odyssey blocking buffer to 1:10,000 and incubated at room temperature for 1 h. Membranes were then washed with tris-buffered saline containing 0.1% Tween-20 for 1 h. The fluorescence signal was detected and analyzed with an Odyssey Clx Imaging system (LI-COR). Quantification of protein levels was performed with ImageJ software (National Institutes of Health, Bethesda, MD).

### In vivo xenograft growth

All animal studies were performed according to a protocol approved by the St. Jude Children’s Research Hospital Institutional Animal Care and Use Committee. We purchased 6-week-old female Crl:NU-*Foxn1*^*nu*^ (*NU/NU*) mice from Charles River Laboratories (Wilmington, MA). The mice were housed with free access to food and water in a room maintained at 22–23 °C with a 12 h light/dark cycle in the St. Jude Animal Resources Center, which is certified by the American Association for Accreditation of Laboratory Animal Care. RD or RH30 doxycycline-inducible *HNRNPH1* shRNA stable cells were suspended in a solution of PBS/matrigel (1:1). The cells (1 × 10^7^ RD or 1 × 10^6^ RH30) were injected subcutaneously. Water containing 0.4 mg/mL doxycycline was delivered immediately after cell transplantation or later, as specified. Water with doxycycline was changed every 2 to 3 days. We used an electronic caliper to measure the length (the greatest longitudinal diameter) and width (the greatest transverse diameter) of tumors. We calculated tumor volume as ${\mathrm{tumor}}\;{\mathrm{volume}} = {\mathrm{length}} \times {\mathrm{width}}^2 \times 0.52$. The investigators were not blinded to the group allocation during the experiment or when assessing the outcome.

### RNA sequencing analysis, volcano plots, gene set enrichment analysis, scatter plots, and GO

Total RNA was isolated from cell lines and prepared by TruSeq. We performed RNA sequencing (RNA-seq) with an Illumina HiSeq 2500 (Illumina, San Diego, CA). Resultant stranded paired-end 100-bp sequences were mapped to the hg19 human genome with the STRONGARM pipeline developed for the Pediatric Cancer Genome Project^34^ and counted with HTSEQ^35^. Statistical testing to determine differential expression was performed in R by using the voom and limma packages. By using our rnapeg in-house tool, exon junction reads were extracted to visualize alternative splicing and select differential junctions and to supplement multivariate analysis of transcript splicing (MATS) 3.0.8 (python 2.7.2)^36^. Results with a false discovery rate less than 5% in MATS analyses were tested for category enrichment with Enrichr^37,38^. Scatterplots, pie charts, and bar charts were produced in STATA 14.2/MP (College Station, TX). Principle component analyses and heat maps were produced with Partek Genomics Suite 6.6 (St Louis, MO). GO analysis was done with Gene Ontology enRIchment anaLysis and vizuaLizAtion tool (Gorilla)^39^.

### Statistical analysis

All results, except RNA-seq and splicing data, were analyzed with GraphPad Prism software (GraphPad Software, La Jolla, CA). For gene expression experiments, data from at least triplicated experiments were quantitatively analyzed by one-way ANOVA with Dunnett multiple comparisons test. For xenograft experiments, data were quantitatively analyzed by Student 2-tailed *t* tests. All data are expressed as the mean ± SD. Sample sizes were chosen according to previous studies that showed statistical significance^40,41^ which also contained information on assay validation and determination of Z’ factor (a measure of statistical effect size)^41^. No data were excluded from the analyses.

### Data availability

All data supporting the results of this study are available within this article and its Supplementary Information. RNA-seq data has been deposited and are available under GEO accession number GSE104559.

## Electronic supplementary material

Supplementary Figure Legends

Table S1

Figure S1

Figure S2

Figure S3

Figure S4

Figure S5

Figure S6

Description of Dataset 1 and 2

Dataset 2

Dataset 1
