# Supplementary material for: HNRNPH1 is required for rhabdomyosarcoma cell growth and survival
Source: Oncogenesis. 2018 Jan 24;7(1):9. doi: 10.1038/s41389-017-0024-4 (PMC5833419; doi:10.1038/s41389-017-0024-4)
Supplement: Supplementary file 1 — Supplementary Figure Legends [file 41389_2017_24_MOESM1_ESM.docx]

**HNRNPH1 is required for rhabdomyosarcoma cell growth and survival**

Yanfeng Li^a#^, Jesse Bakke^a#^, David Finkelstein^b^, Hu Zeng^c, d^, Jing Wu^a^, Taosheng Chen^a,*^

^a^*Department of Chemical Biology and Therapeutics, St. Jude Children’s Research Hospital, Memphis TN, USA*

^b^*Department of Computational Biology, St. Jude Children’s Research Hospital, Memphis TN, USA*

^c^*Department of Immunology, St. Jude Children’s Research Hospital, Memphis TN, USA*

^d^*Current: Division of Rheumatology, Department of Medicine, Department of Immunology, Mayo Clinic, Rochester MN, USA*

^#^These authors contributed equally to this work

*Corresponding author. St. Jude Children’s Research Hospital, 262 Danny Thomas Place, MS-1000, Memphis, TN 38105, USA. Tel: 901 595 5937; Fax: 901 595 5715; E-mail address: [taosheng.chen@stjude.org](mailto:taosheng.chen@stjude.org) (T. Chen)

This work was supported by ALSAC and the National Institutes of Health [grant numbers RO1-GM110034, R35-GM118041, and P30-CA21765].

**Running title: HNRNPH1 in rhabdomyosarcoma**

**Supplementary Figure Legends**

**Supplementary Fig. S1, related to Fig. 1.** Quantitative PCR to detect the mRNA levels of HNRNPH1 in differentiated LHCN, SKMC, and HSMM cells, and several patient derived xenografts (PDX). PDX samples marked in red are ARMS and blue are ERMS. Each data point is an average of three replicates for each patient sample and the data is presented as relative expression.

**Supplementary Fig. S2, related to Fig. 1 and Fig 2.** (A) Images obtained with Incucyte Zoom at 4 days post transfection of 3 individual *HNRNPH1* siRNAs (si#1, si#2, and si#3), as well as siControl (siCon) in RMS (RD, RH30 and RH41) cells. (B) Gene Ontology (GO) analysis of differentially expressed genes in RD, RH30, and RH41. (C-D) Venn diagram of differentially expressed genes that have a greater than two-fold change in both siRNA #1 and siRNA#2 (HNRNPH1) for each cell type. Listed are the genes in common among all three cell lines.

**Supplementary Fig. S3, related to Fig. 3. (**A–C) Quantitative RT-PCR of *CDK2*, *CDK4*, and *CDK6* expression were analyzed 48 h post transfection of *HNRNPH1* siRNAs in (A) RD, (B) RH30, and (C) RH41 cells. Data are expressed relative to siCon cells (mean ± SD, n = 3). **P* < 0.05; ***P* < 0.01; ****P* < 0.001; *****P* < 0.0001.

**Supplementary Fig. S4, related to Fig. 4. (**A–B) Immunoblot analysis of whole-cell lysates prepared from (A) RD and (B) RH30 cells used in xenograft assays (Fig. 4A and B). The cells were induced with doxycycline (DOX) for 4 or 7 days and subjected to immunoblot analysis with antibodies against HNRNPH1 and β-actin. Quantification for HNRNPH1 shown below the gels and is normalized to actin. (C–D) Mice were treated with 0.4 mg/mL DOX in their drinking water after either 12 (RH30) or 25 (RD) days after cell transplantation, as indicated. (C) RD and (D) RH30 tumor volumes were measured at the indicated days after cell transplantation. (E) RD and (F) RH30 xenografts were collected and weighed at the end of the experiment. **(**G–H) Immunoblot analysis of cell lysates prepared from the xenografts shown in Figure 4G (S4G upper), S4E (S4G lower), 4H (S4H upper) and S4F (S4H lower) were performed with antibodies against HNRNPH1 and β-actin. Quantification is an average of each group normalized to actin. (I) Tumor volume measurements and western blot analysis (J) from a second cohort (same study design as described for Fig. S4D). Day 12 is the first day of doxycycline treatment and mice were sacrificed at days 12, 14, and 17 to measure tumor volumes. Western blot analysis in (J) show two representative plko vector control tumors, and both sh#1 tumors (Day 17) are a mixture of two separate tumors (four in total) that were combined due to the small tumor sizes. Quantification is shown normalized to actin.

**Supplementary Fig. S5, related to Fig. 5.** The list of genes analyzed in Fig. 5C, according to RMS cell type.

**Supplementary Fig. S6, related to Fig. 6. (**A) Schematic of different *CTNNB1* gene splicing forms and the locations of the primers used for quantitative RT-PCR to detect each of them separately. (B) Quantitative RT-PCR analysis of each splicing form of the *CTNNB1* gene in RMS cells**.** (C–E) Quantitative RT-PCR analysis of total *CTNNB1* gene expression and its 2 most abundant splicing forms 48 h post transfection of *HNRNPH1* siRNAs in (C) RD, (D) RH30, and (E) RH41cells. (F) Schematic of different *MDM4* gene splicing forms and the locations of the primers used for RT-PCR to detect the changes in exon junctions determined by RNA-seq. (G) Semi-quantitative RT-PCR analysis of the *MDM4* exon junction 48 h post transfection of *HNRNPH1* siRNAs in RMS cells. The upper band is the splicing form with inclusion of part of intron 1 region. The lower band is wild type splicing form. The bottom graphic depicts the sequencing results of the alternative splicing caused by *HNRNPH1* siRNAs.
