# Supplementary material for: HNRNPH1 is required for rhabdomyosarcoma cell growth and survival
Source: Oncogenesis. 2018 Jan 24;7(1):9. doi: 10.1038/s41389-017-0024-4 (PMC5833419; doi:10.1038/s41389-017-0024-4)
Supplement: Supplementary file 2 — Table S1 [file 41389_2017_24_MOESM2_ESM.docx]

**Supplementary Table S1**

Primers used for *CTNNB1* gene quantitative RT-PCR and *MDM4* semi-quantitative RT-PCR.

| Primer | Sequence | Product length |
| --- | --- | --- |
| CTNNB1all-f1 | AGCCACAAGATTACAAGAAACGG | 198 bp |
| CTNNB1all-r1 | TCCATACCCAAGGCATCCTG |  |
| CTNNB1 NM1904Exon15f | CTCCAGGTGACAGCAATCAG | 163 bp |
| CTNNB1 NM1904Exon15r | TTACAAATAGCCTAAACCACTCCC |  |
| CTNNB1 NM1098209Exon15-16f | GACCTGTAAATCATCCTTTAGCTG | 194 bp |
| CTNNB1 NM1098209Exon16r | TTGTATTGTTACTCCTCGACCA |  |
| CTNNB1 NM1098210Exon16-16f | GACCTGTAAATCATCCTTTAGGAG | 185 bp |
| CTNNB1 NM1098210Exon16r | CTACTTCAAAGCAAGCAAAGTC |  |
| CTNNB1 NM1330729Exon2-3f | CAATGGCTACTCAAGGCTACC | 169 bp |
| CTNNB1 NM1330729Exon4r | CATGTCCAACTCCATCAAATCAG |  |
| MDM4-Exon1f | GTGTGGGAGGCCGGAAGTTG | 243 bp |
| MDM4-Exon2r | TGATTGATTTGTCCAGGAGAGATCC |  |
