# Supplementary figures and images for: HNRNPH1 is required for rhabdomyosarcoma cell growth and survival
Source: Oncogenesis. 2018 Jan 24;7(1):9. doi: 10.1038/s41389-017-0024-4 (PMC5833419; doi:10.1038/s41389-017-0024-4)

Figure S1

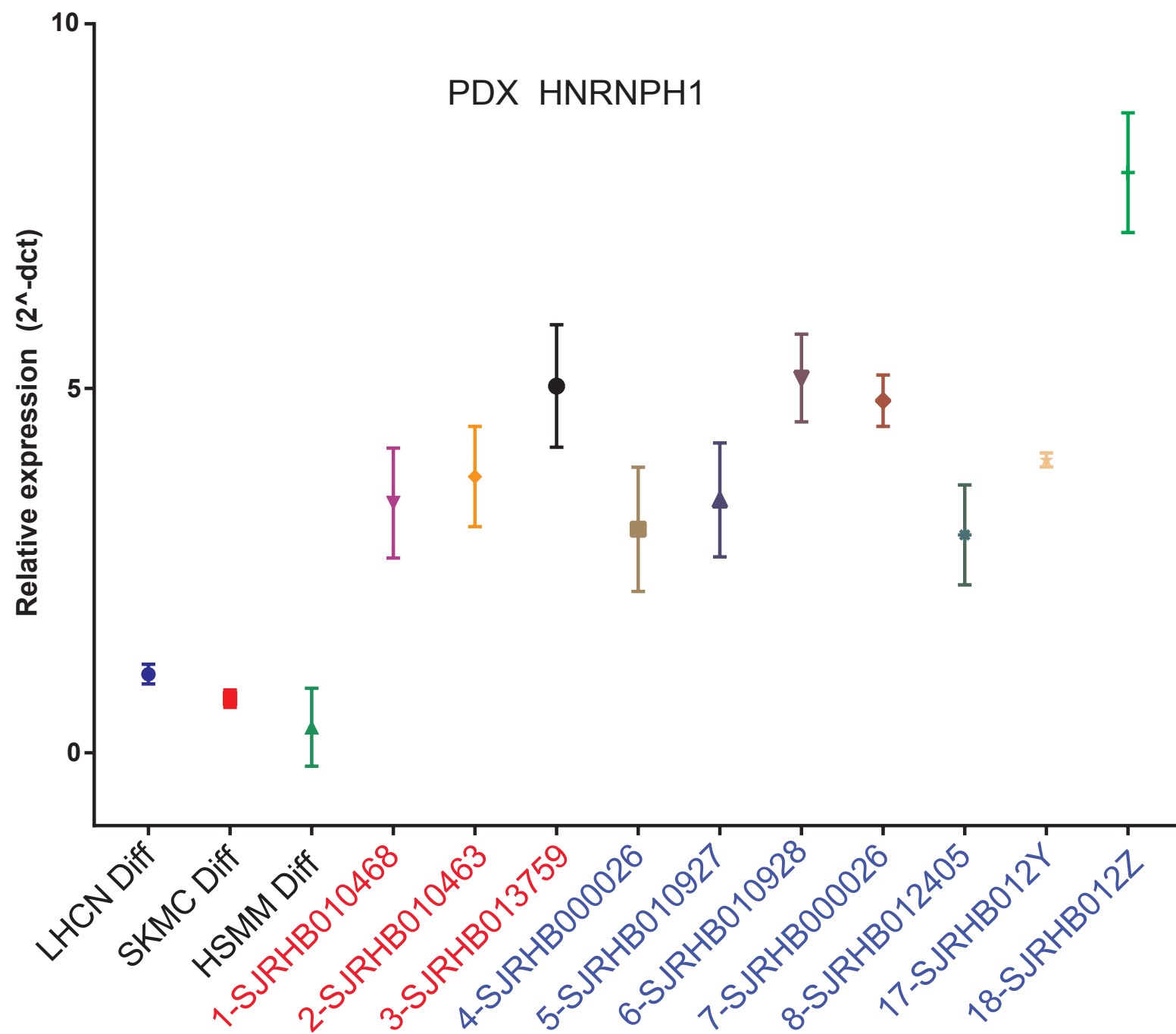

Supplement: Supplementary file 3 — Figure S1 [file 41389_2017_24_MOESM3_ESM.pdf]

Figure S3

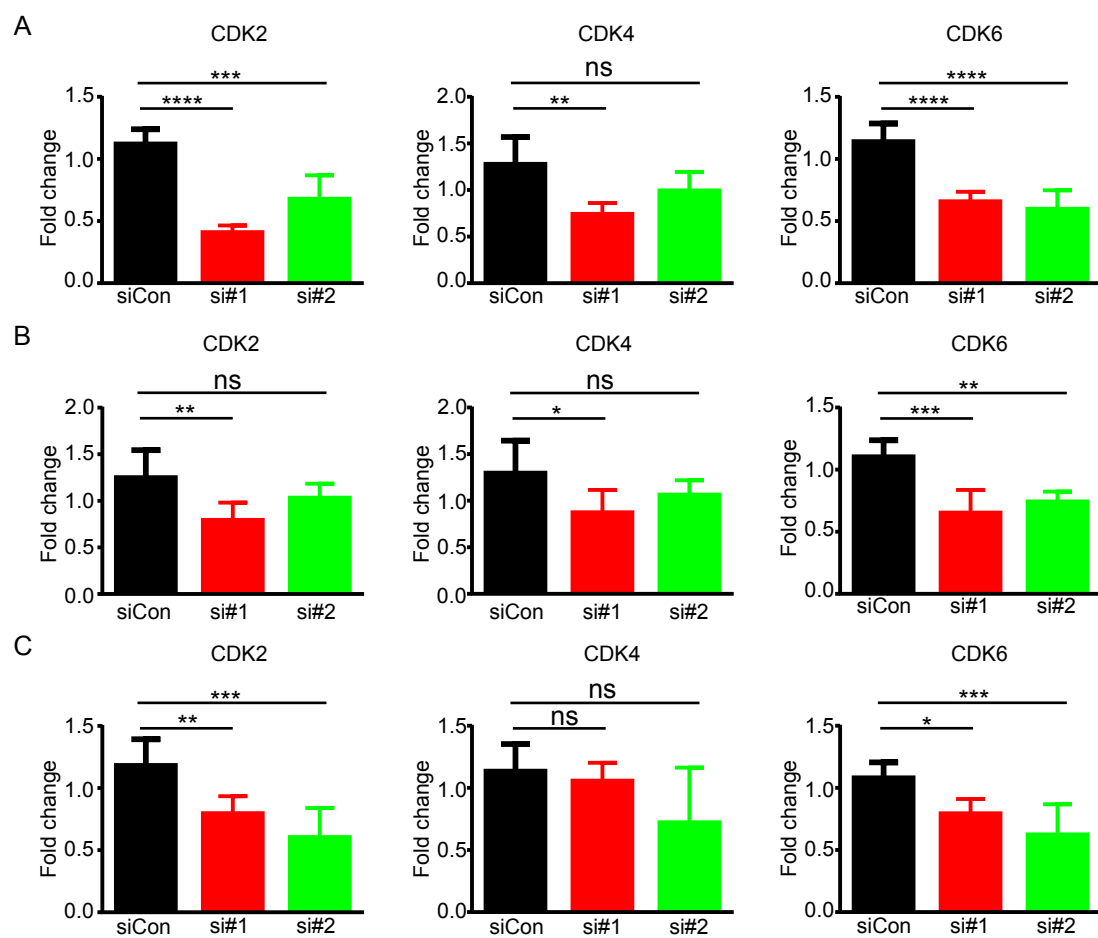

Supplement: Supplementary file 5 — Figure S3 [file 41389_2017_24_MOESM5_ESM.pdf]

Figure S4

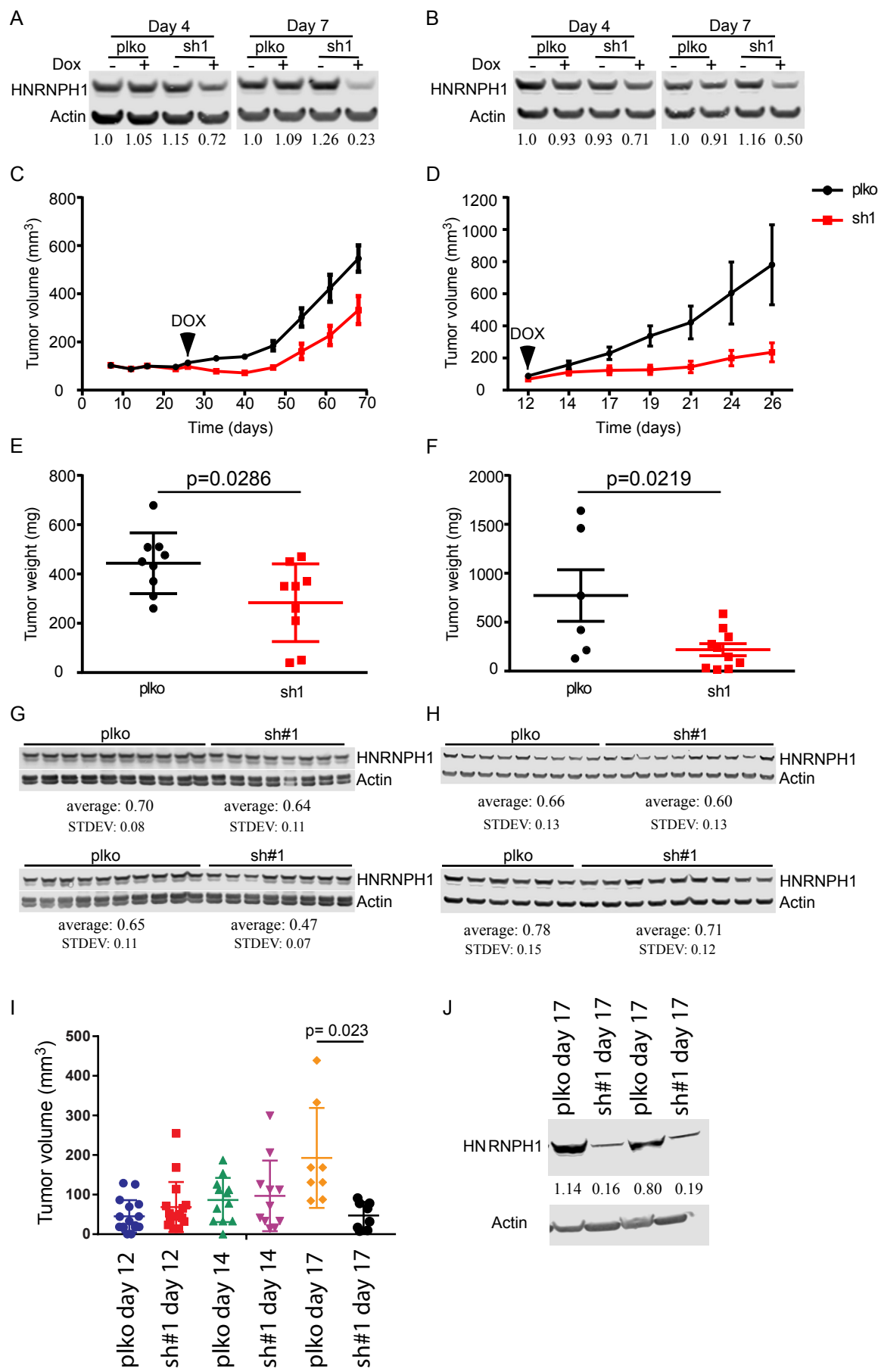

Supplement: Supplementary file 6 — Figure S4 [file 41389_2017_24_MOESM6_ESM.pdf]

Figure S5

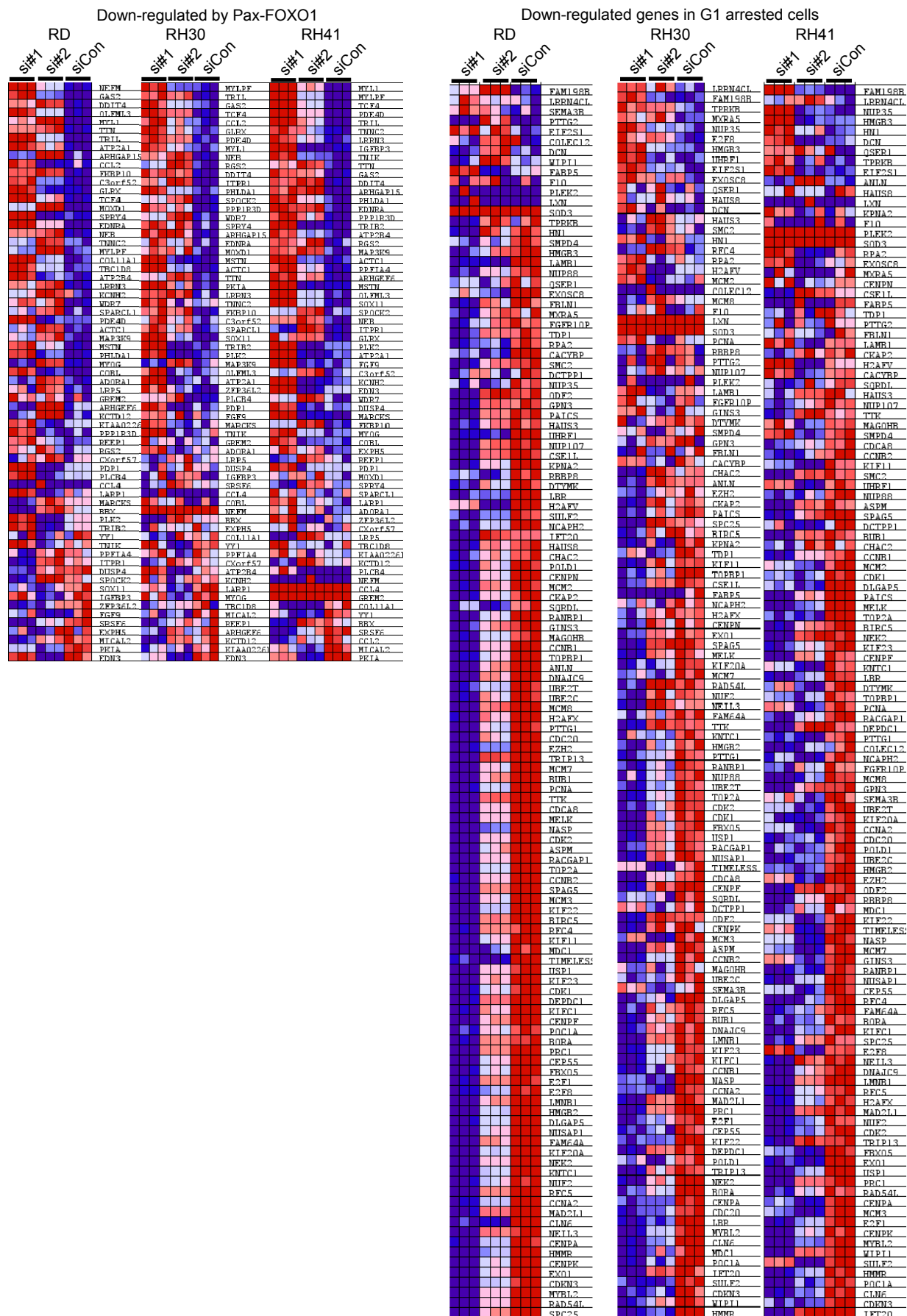

Supplement: Supplementary file 7 — Figure S5 [file 41389_2017_24_MOESM7_ESM.pdf]

Figure S6

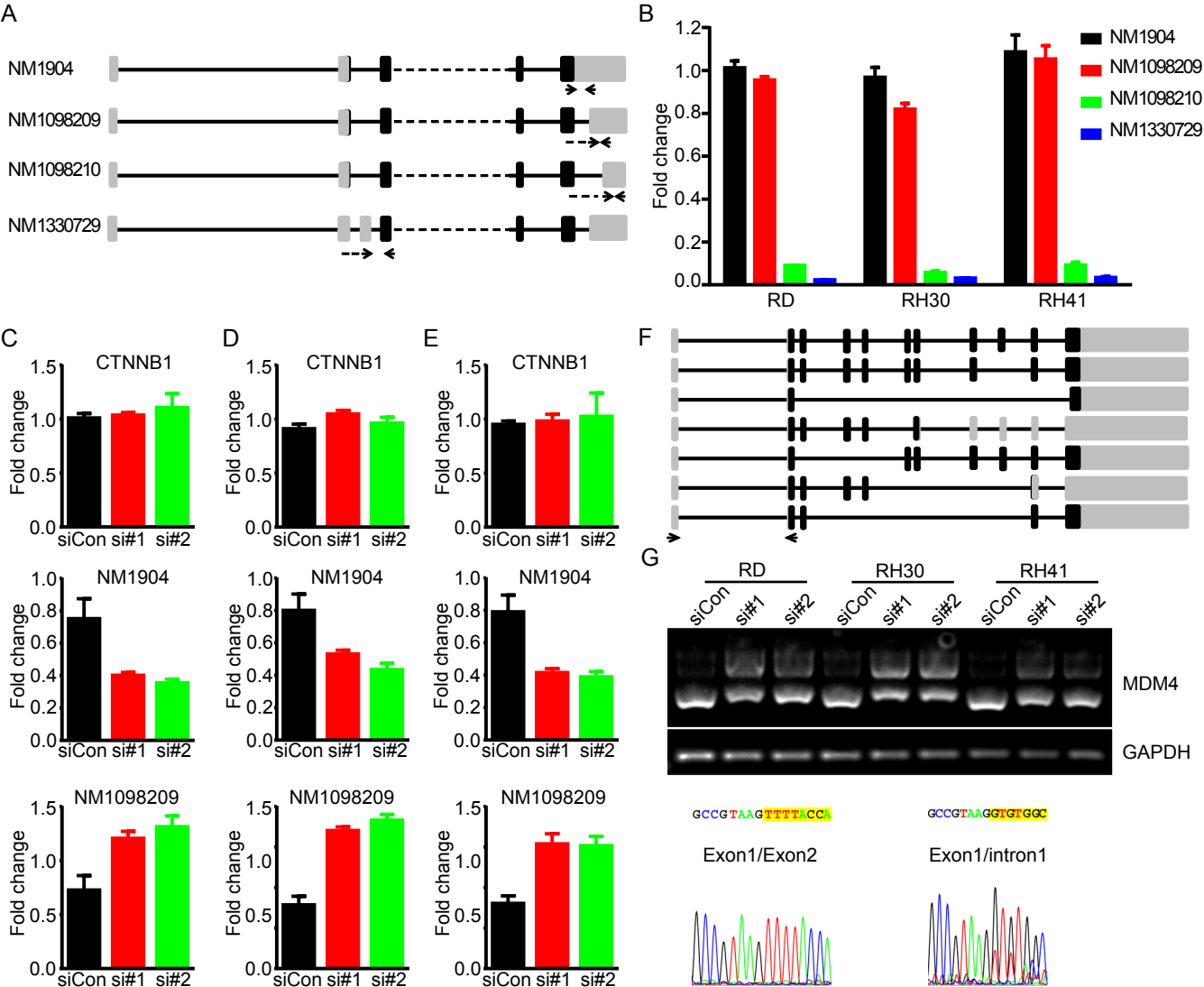

Supplement: Supplementary file 8 — Figure S6 [file 41389_2017_24_MOESM8_ESM.pdf]
