# Supplementary material for: HNRNPH1 is required for rhabdomyosarcoma cell growth and survival
Source: Oncogenesis. 2018 Jan 24;7(1):9. doi: 10.1038/s41389-017-0024-4 (PMC5833419; doi:10.1038/s41389-017-0024-4)
Supplement: Supplementary file 4 — Figure S2 [file 41389_2017_24_MOESM4_ESM.pdf]

Figure S2

A.

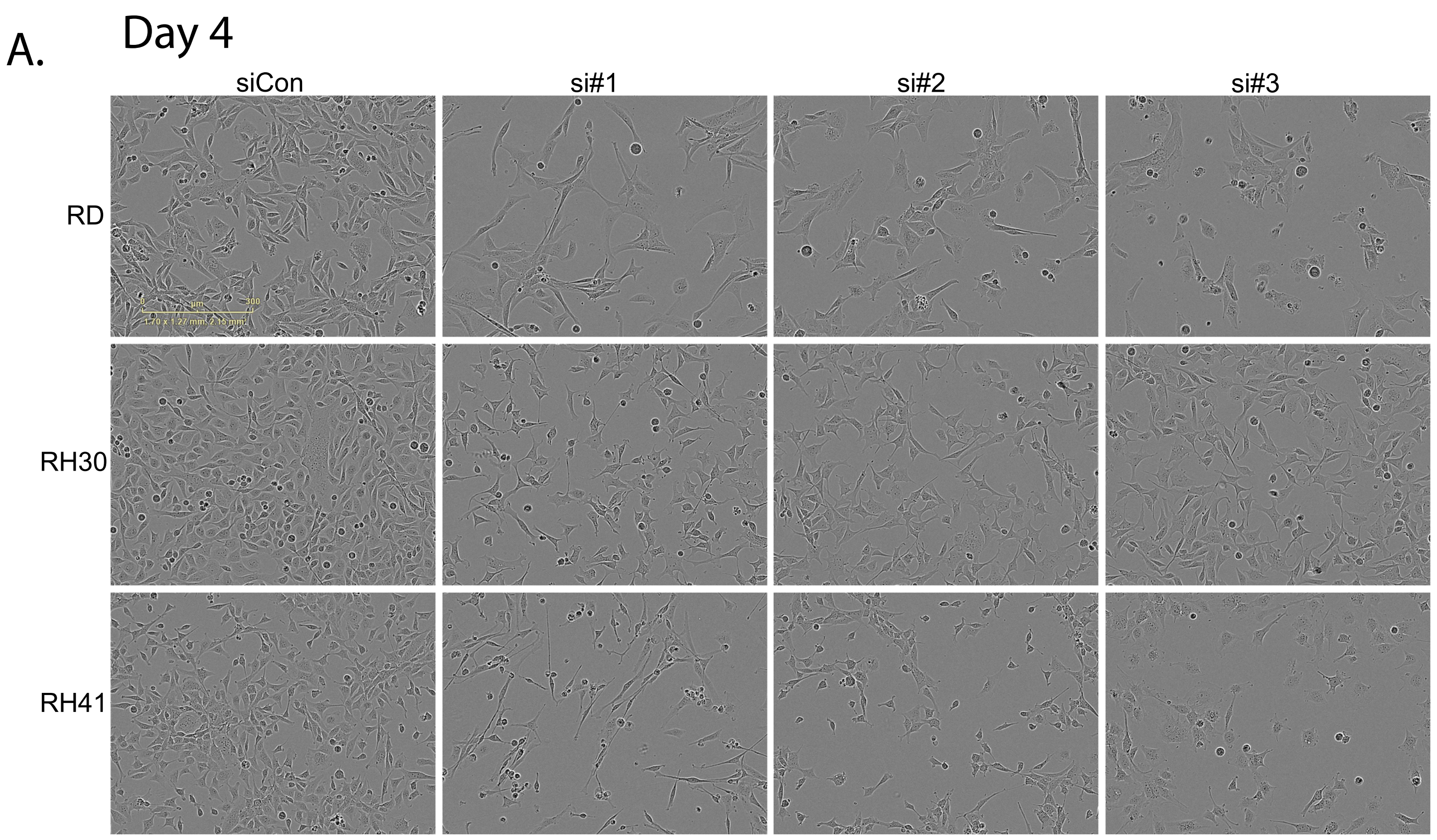

B.

RD Down

Go Term	Description	P-Value	FDR q-Value
GO: 0006334	Nucleosome Assembly	5E-40	4.65E -36
GO: 0065004	Protein-DNA Complex Assembly	7.7E -37	2.39E -33
GO: 0000183	Cell Cycle Process	8.65E -23	2.57E -20

RD Up

Go Term	Description	P-Value	FDR q-Value
GO: 0010469	Regulation of Receptor Activity	1.13E -6	1.14E -2
GO: 0042391	Regulation of Membrane Potential	3.23E -5	5.38E -2
GO: 0007155	Cell Adhesion	3.85E -5	4.39E -2

Rh30 Down

Go Term	Description	P-Value	FDR q-Value
GO: 0006952	Defense Response	8.18E -5	7.44E -1
GO: 0051591	Response to cAMP	1E -4	4.55E -1
GO: 0048731	System Development	1.15E -4	3.47E -1

Rh30 Up

Go Term	Description	P-Value	FDR q-Value
GO: 0044057	Regulation of System Process	1.64E -6	1.6E -2
GO: 0006936	Muscle Contraction	7.48E -6	3.69E -2
GO: 0003012	Muscle System Process	3.79E -5	2.87E -2

Rh41 Down

Go Term	Description	P-Value	FDR q-Value
GO: 0006334	Nucleosome Assembly	1.02E -12	9.37E -9
GO: 0065004	Protein-DNA Complex Assembly	2.12E -12	6.48E -8
GO: 0006952	Defense Response	2.41E -8	2.61E -5

Rh41 Up

Go Term	Description	P-Value	FDR q-Value
GO: 0006936	Muscle Contraction	3.05E -5	2.62E -1
GO: 0003008	System Process	8.47E -5	3.66E -1
GO: 0003012	Muscle System Process	9.39E -5	5.32E -1

C.

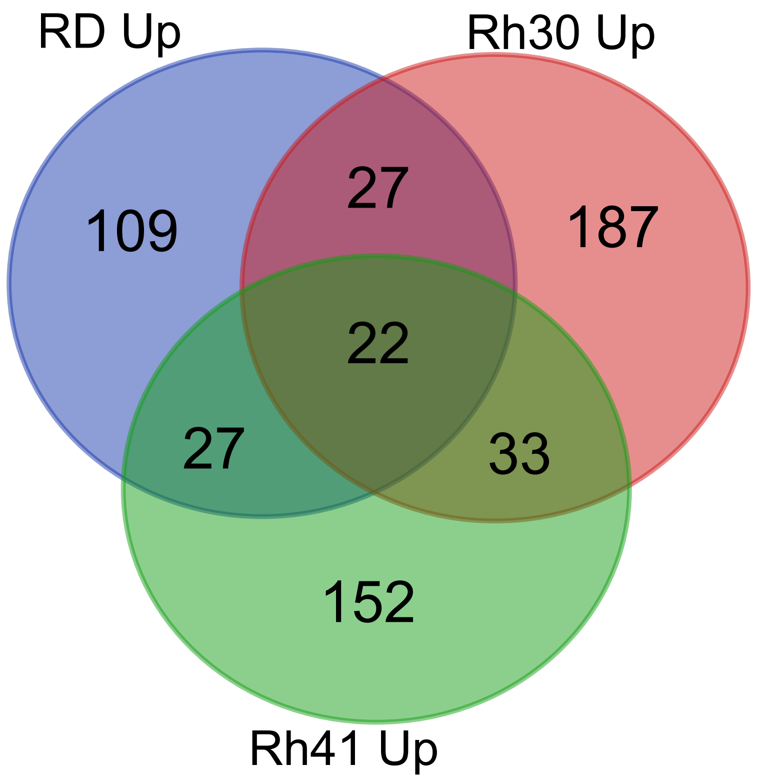

SPAG17, PTPN22, AC006538.1, DHH, KRTCAP3, KCDN1, AC093627.10, OVGP1, LAG3, PIWIL2, PAPPA, LY6G6C, CNKSR1, SLC22A1, RP4-751H13.7, HRC, LRRC17B, FAM78A, CPNE9, CTB-109A12.1, CTB-47B11.3, SCN3BRD

D.

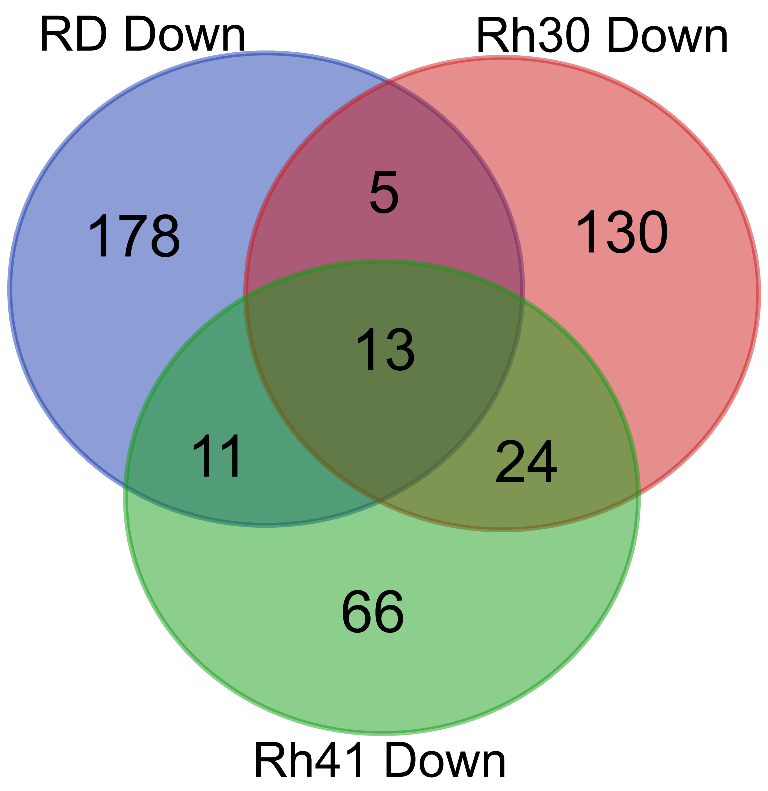

HFE, CCNA1, HNRNPH1, NUCKS1, RP11-22P6.3, ICT1, AC016629.8, RBM3, E2F5, PLCB3, TFAP2A-AS1, C18orf56, PAK2RD
