# Supplementary material for: HNRNPH1 is required for rhabdomyosarcoma cell growth and survival
Source: Oncogenesis. 2018 Jan 24;7(1):9. doi: 10.1038/s41389-017-0024-4 (PMC5833419; doi:10.1038/s41389-017-0024-4)
Supplement: Supplementary file 9 — Description of Dataset 1 and 2 [file 41389_2017_24_MOESM9_ESM.docx]

**Dataset 1: List of genes with altered expression by HNRNPH1 siRNA in RMS cells**

**Dataset 2: List of genes with altered exon junction** **by HNRNPH1 siRNA in RMS cells**
